# The Host Range of Gammaretroviruses and Gammaretroviral Vectors Includes Post-Mitotic Neural Cells

**DOI:** 10.1371/journal.pone.0018072

**Published:** 2011-03-28

**Authors:** Xiu-Huai Liu, Wenqin Xu, Jill Russ, Lee E. Eiden, Maribeth V. Eiden

**Affiliations:** 1 Section on Molecular Neuroscience, Laboratory of Cellular and Molecular Regulation, National Institute of Mental Health, National Institutes of Health, Bethesda, Maryland, United States of America; 2 Section on Directed Gene Transfer, Laboratory of Cellular and Molecular Regulation, National Institute of Mental Health, National Institutes of Health, Bethesda, Maryland, United States of America; George Mason University, United States of America

## Abstract

**Background:**

Gammaretroviruses and gammaretroviral vectors, in contrast to lentiviruses and lentiviral vectors, are reported to be restricted in their ability to infect growth-arrested cells. The block to this restriction has never been clearly defined. The original assessment of the inability of gammaretroviruses and gammaretroviral vectors to infect growth-arrested cells was carried out using established cell lines that had been growth-arrested by chemical means, and has been generalized to neurons, which are post-mitotic. We re-examined the capability of gammaretroviruses and their derived vectors to efficiently infect terminally differentiated neuroendocrine cells and primary cortical neurons, a target of both experimental and therapeutic interest.

**Methodology/Principal Findings:**

Using GFP expression as a marker for infection, we determined that both growth-arrested (NGF-differentiated) rat pheochromocytoma cells (PC12 cells) and primary rat cortical neurons could be efficiently transduced, and maintained long-term protein expression, after exposure to murine leukemia virus (MLV) and MLV-based retroviral vectors. Terminally differentiated PC12 cells transduced with a gammaretroviral vector encoding the anti-apoptotic protein Bcl-xL were protected from cell death induced by withdrawal of nerve growth factor (NGF), demonstrating gammaretroviral vector-mediated delivery and expression of genes at levels sufficient for therapeutic effect in non-dividing cells. Post-mitotic rat cortical neurons were also shown to be susceptible to transduction by murine replication-competent gammaretroviruses and gammaretroviral vectors.

**Conclusions/Significance:**

These findings suggest that the host range of gammaretroviruses includes post-mitotic and other growth-arrested cells in mammals, and have implications for re-direction of gammaretroviral gene therapy to neurological disease.

## Introduction

With the identification of a gammaretrovirus, xenotropic murine leukemia virus-related virus, XMRV, as an emerging human pathogen [1], [2], and the use of gammaretroviral vectors in gene therapy for human disease [3], examining the determinants for host cell range of gammaretroviruses and gammaretroviral vectors is of great public health significance. Growth-arrested cells are reported to be susceptible to infection by lentiviruses and lentiviral vectors but not gammaretroviruses [4], [5] or gammaretroviral vectors [6], [7]. This has been attributed to the ability of retroviruses of the lentiviral genus to cross an intact nuclear membrane, owing to unique features of their preintegration complexes ([8], [9], [10], [11], [12], [13], [14], also reviewed by Goff, and Suzuki and Craigie [15], [16]).

It has been proposed that lentiviral vectors have an advantage over simple retroviral vectors in their ability to deliver genes to nondividing cells because of unique genetic components intrinsic to lentiviruses that allow the HIV pre-integration complex (PIC) to cross the nuclear envelope in non-dividing cells. Third-generation lentiviral vectors that retain the ability to infect neurons contain only the HIV genes gag, pol, and rev, eliminating Vpr, Vpu and Tat, as candidate factors essential for the transduction of neurons and other non-dividing cells. The matrix component and other gag proteins remain in third-generation lentiviral vectors. Matrix was the first component implicated in nuclear import of HIV PICs into the nucleus of non-dividing cells. Matrix was shown to contain a short stretch of amino acid residues similar to nuclear localization signals of cellular proteins, and to localize matrix-fused cytosolic proteins to the nucleus [17], [18]. However, subsequent studies have not substantiated this role for matrix [16]. Furthermore, matrix present in HIV particles does not localize to the nucleus [19]. So far, no factors have been identified that confer the ability of lentiviruses to infect non-dividing cells onto gammaretroviral vectors [16]. Reports on the susceptibility of non-dividing human monocytes or macrophages to gammaretroviruses have suggested that they can be transduced at efficiencies as high as 25% with Friend murine leukemia virus (F-MLV)-based vectors harboring a rat VL30 packaging site [20]. On the other hand, macrophages are said to harbor restrictive blocks to murine leukemia virus (MLV) infection that can be overcome by incorporating Vpx of HIV2/SIV into an MLV-based vector [21]. Thus, it has so far not been possible to verify the hypothesis, through transfer of discrete lentiviral elements to gammaretroviral vectors, that unique and distinct lentiviral components confer the ability to transduce post-mitotic cells.

The lack of consensus as to which retroviral components are required for integration into post-mitotic cells caused us to embark on a more detailed comparison of the structural requirements underlying the presumed differential ability of gammaretroviruses and retroviral vectors, compared to lentiviruses and lentiviral vectors, to transduce, and integrate into the genomes of, post-mitotic cells. In the process, we made the serendipitous discovery, using viruses with a green fluorescent protein marker (GFP), that infection of neurons, as well as growth arrested neuroendocrine cells, occurs with murine leukemia replication-competent gammaretroviruses and MLV-based vectors at an efficiency similar to that of lentiviral vectors, and sufficient to confer transgene mediated phenotypes to cultured cells.

## Results

### Comparison of gammaretroviral and lentiviral vector transduction of proliferating and differentiated PC12 cells

**Figure 1:**
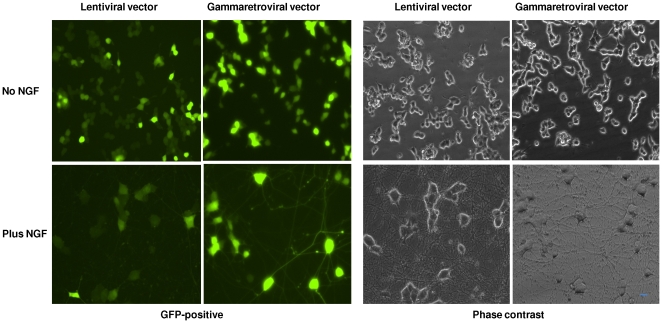
Comparison of transduction efficacy between lentiviral and gammaretroviral vectors in proliferating and differentiated PC12 cells. PC12 cells were grown in 4-well Matrigel-coated chamber slides and treated with 100 ng/ml NGF for 5 days before viral infection (Plus NGF) or with vehicle alone for one day before viral infection (no NGF). Cells were exposed to an identical titer (9.3×10^5^ infectious lentiviral or RT43.2GFP gammaretroviral particles/chamber). 7 days after infection, the cells were photographed at 10× with FITC filter (GFP-positive, virally transduced cells) or phase contrast microscopy (all cells). Transduction efficiency was similar for both types of virus particle, and for both undifferentiated and differentiated cells. Scale bar = 20 µm.

**Figure 2:**
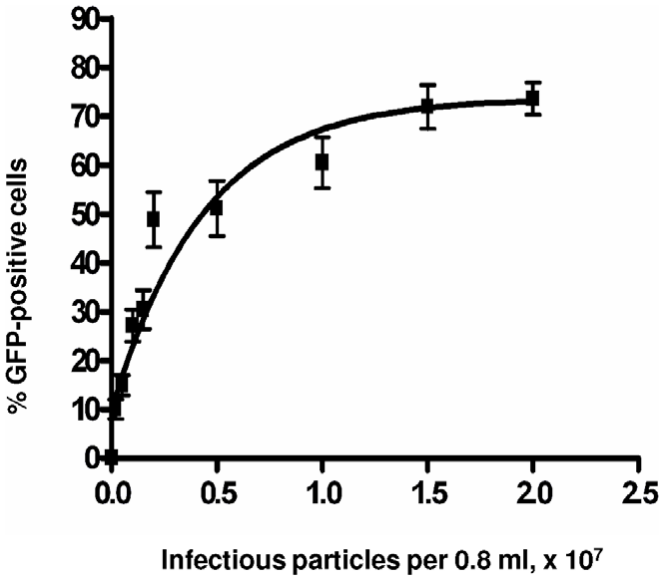
RT43.2GFP gammaretroviral vector transduction into differentiated PC12 cells: effect of increased viral titer. After five days of NGF treatment, differentiated PC12 cells grown on 4-well chamber slides coated with Matrigel were exposed to concentrations of viral particles ranging from 2.0×10^5^ to 2.0×10^7^ RT43.2GFP vector particles in the presence of NGF. Three days after exposure to vectors, cells were photographed with a 20× objective, using a FITC or DAPI filter, after addition of DAPI. Transduction efficiency is expressed as the mean of the percentage of DAPI-positive cells expressing GFP (ordinate) from several fields for each transduction experiment, for different titers of vector particles (abscissa).

PC12 cells are a cell line derived from a rat pheochromocytoma of the adrenal medulla [22] that extend neurites and undergo growth arrest in the G_1_ phase of the cell cycle when treated with nerve growth factor (NGF) [23], [24], [25]. We initiated our investigation of differential gammaretroviral and lentiviral vector transduction of non-dividing cells by comparing transduction of PC12 cells by the gammaretroviral vector RT43.2GFP and the lentiviral vector RRL-SIN-18 either before or after differentiation with NGF. PC12 cells were exposed to vesicular stomatitis virus (VSV)-enveloped MLV-based or HIV-based vectors (schematically shown in Figure S1) produced in 293T cells and titered on murine *Mus dunni* tail fibroblasts (MDTF cells) [26]. We used approximately 9.3×10^5^ infectious viral particles (as determined by titration on a monolayer of MDTF cells) per chamber (each containing 2.7×10^4^ cells as initially plated). This represents a multiplicity of infection (MOI; virus particles per target cell) on the PC12 cells of about 30. This MOI was chosen so that 100% of the PC12 cells would be transduced, if transduction was as efficient as in MDTF cells (i.e. PC12 cells were ∼5% confluent at initiation of exposure to virus, so that an MOI of 20 would be predicted to transduce all cells). Transduction with both types of vector was about equivalent both in proliferating (Figure 1, No NGF) and differentiated (Figure 1, Plus NGF) cells and, based on the calculated MOI and percent confluence, about 50% as efficient for both types of vector particles as on MDTF cells. We speculate that the slight loss in transduction efficiency for both lenti- and gammaretroviral vectors in either undifferentiated or differentiated PC12 cells, relative to MDTF cells, is attributable in part to the culturing of PC12 cells in Matrigel, causing a partial barrier to efficient vector-cell contact. In support of this, increasing MOI substantially beyond 30 increased transduction efficiency to a maximum of about 75% (Figure 2), suggesting that a proportion of the cells are inaccessible to virus in the Matrigel configuration. We also note here that green fluorescence following transduction with either lentiviral or gammaretroviral vectors develops progressively within 2–3 days after transduction, consistent with viral integration, gene transcription, and translation of GFP mRNA, and ruling out the possibility of pseudotransduction through GFP expression via translation of unintegrated viral RNA [27].

### Characteristics of gammaretrovirus/gammaretroviral vector transduction of differentiated PC12 cells

**Figure 3:**
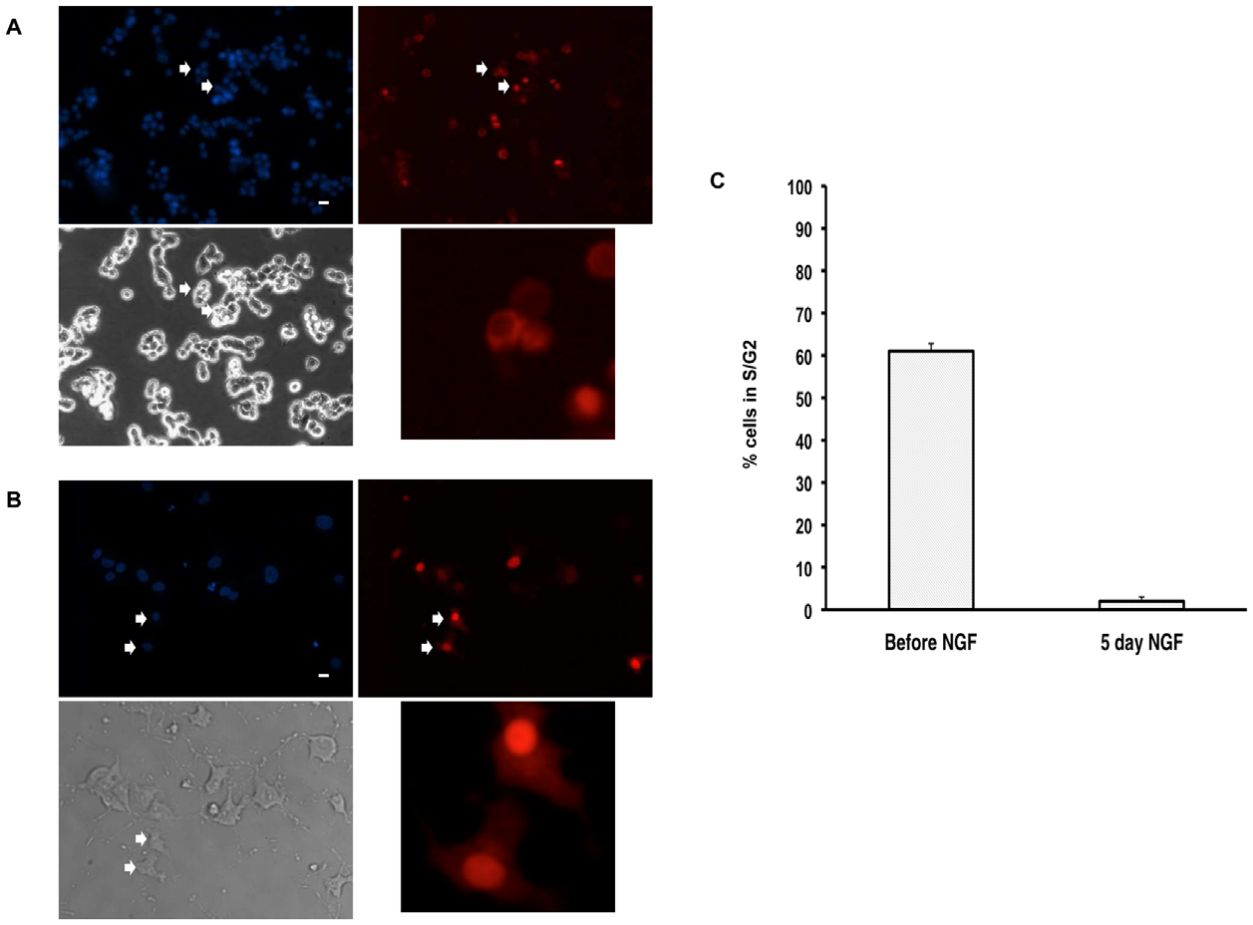
Quantification of S/G2 cells in untreated and NGF-treated LNCX-HDHBc-tdimer2-transduced PC12 cells. LNCX-HDHBc-tdimer2-transduced PC12 cells were plated into Matrigel-coated 4-well chamber slides, and left untreated for two days (No NGF; Figure 3A) or treated with 100 ng/ml NGF for 5 days to allow full differentiation (Plus NGF; Figure 3B). Photodocumentation was at 20× after staining with DAPI for nuclear visualization by epifluorescence (blue), by phase contrast for cell visualization (black-and-white) or for nuclear or cytoplasmic HDHBc-tdimer2 visualization by epifluorescence (red). **A**. Arrows indicate an example of one cell (upper) in which fluorescence is excluded from the nucleus (S1 phase), and one (lower) in which fluorescence is restricted to the nucleus (G1 phase) in proliferating (No NGF) PC12 cells. Scale bar = 10 µm. Panel at lower right presents higher magnification of cells indicated by arrowheads immediately above. **B**. Arrows indicate two cells in which fluorescence is restricted to the nucleus (G1 phase) in differentiated (Plus NGF) PC12 cells. Scale bar = 10 µm. Panel at lower right presents higher magnification of cells indicated by arrowheads immediately above. **C**. Percentage of cells with nuclear exclusion (versus sum of nuclear-excluded and nuclear-localized cells) is plotted for No NGF and Plus NGF conditions.

Transduction of differentiated PC12 cells by the gammaretroviral vector at an efficiency comparable to that of lentiviral vectors cannot be explained by transduction of a background of cells that is still proliferating after 5 days of treatment with NGF. It has previously been shown [28], [29] and was subsequently confirmed by us in the PC12 cell line used here (data not shown) that NGF causes a progressive loss of BrdU incorporation from close to 100% in unsynchronized, proliferating cells to 0–20% after 5 days of exposure to NGF. Thus, a background of proliferating cells could not account for an equivalent efficiency of transduction at a fixed viral input, as observed here (Figure 1). Retroviral vectors are said not be amenable to transduction of cells in G1 phase because it is during this time that the nuclear membrane is intact, and the pre-integration complex cannot be formed in, or transported to, the nucleus. We examined the possibility that although they are growth arrested, PC12 cells are not completely arrested in a state (G1) in which the nuclear membrane is intact. Recently, Hahn et al. devised an indicator for growth arrest in G1 [30], consisting in the C-terminus of the HDHB helicase, which is contained exclusively in the nucleus in G1 and is exclusively cytoplasmic in S1/G2, fused to tdimer2, an RFP ‘intramolecular’ red fluorescent protein dimer developed by Campbell et al. [31]. We transferred the expression cassette for this biological reporter into a LNCX vector that provides expression of aminoglycoside phosphotransferase conferring resistance to the antibiotic G418. This construct was packaged as described for RT43.2GFP, transduced into PC12 cells, and G418-resistant cells selected by growth in medium containing 0.8 mg/ml G418. This cell line was used to analyze the percentage of cells in G1 phase before and after treatment with NGF for five days. As shown in Figure 3, most of the cells in the culture (more than 98%) were in G1 phase after five days of NGF treatment. Although proliferating cells also cycle through the G1 phase, the doubling time for PC12 cells (greater than 48 hours, see Ravni et al., 2008 [32]) allows the conservative estimate that PC12 cells treated with NGF for five days offer no more than 5% of the total population not in G1 phase over the period of time of viral exposure and transduction. Thus, selective transduction of cells not in G1 phase cannot explain the ability of gammaretroviral vectors to transduce up to 80% of NGF-treated PC12 cells in culture.

**Figure 4:**
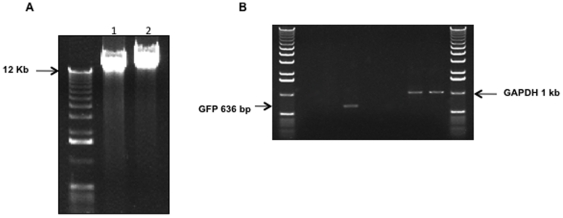
Detection of GFP-coding sequences within genomic DNA prepared from terminally differentiated PC12 cells transduced with RT43.2GFP vectors. **A**. Representative gel picture showing genomic DNA fragment with size >10 Kb was used for PCR analysis. Differentiated PC12 cells (5 days with NGF treatment) were exposed to RT43.2GFP vectors for 3 days, and then genomic DNA was isolated and run on a 0.8% agarose gel to purify DNA fragment with size >10 Kb. (lane 2). Non-transduced PC12 cells were used as a negative control as represented by lane 1. **B**. PCR analysis. The primer pair used for lane 1 (H_2_O), lane 2 (control PC12 genomic DNA) and lane 3 (differentiated gel purified transduced PC12 genomic DNA) generated a GFP fragment of 636 bp in lane 3. Control primers specific for GAPDH were used for samples in lanes 5–7. Lanes 5 is an H_2_O control, lane 6 contains DNA purified from control PC12 cells and lane 7 contains gel purified DNA from transduced differentiated PC12 cells; lane 4 is an empty spacer lane.

A second possible explanation for the ability of gammaretroviral vectors to transduce G1-arrested PC12 cells and express protein from the viral vector's promoter is that the vector RNA genome gains access to the cytoplasm, is reverse-transcribed, and that GFP can in turn be transcribed from this proviral extranuclear gene. Although this seems unlikely from a cell biological perspective, we nevertheless isolated genomic DNA from transduced differentiated PC12 cells and subjected this to PCR amplification to assay for integrated vector DNA. Integrated viral DNA was found in differentiated PC12 cells exposed to RT43.2GFP, with copy number qualitatively similar to rat GAPDH, as judged by comparison with GAPDH reference gene amplification (Figure 4).

**Figure 5:**
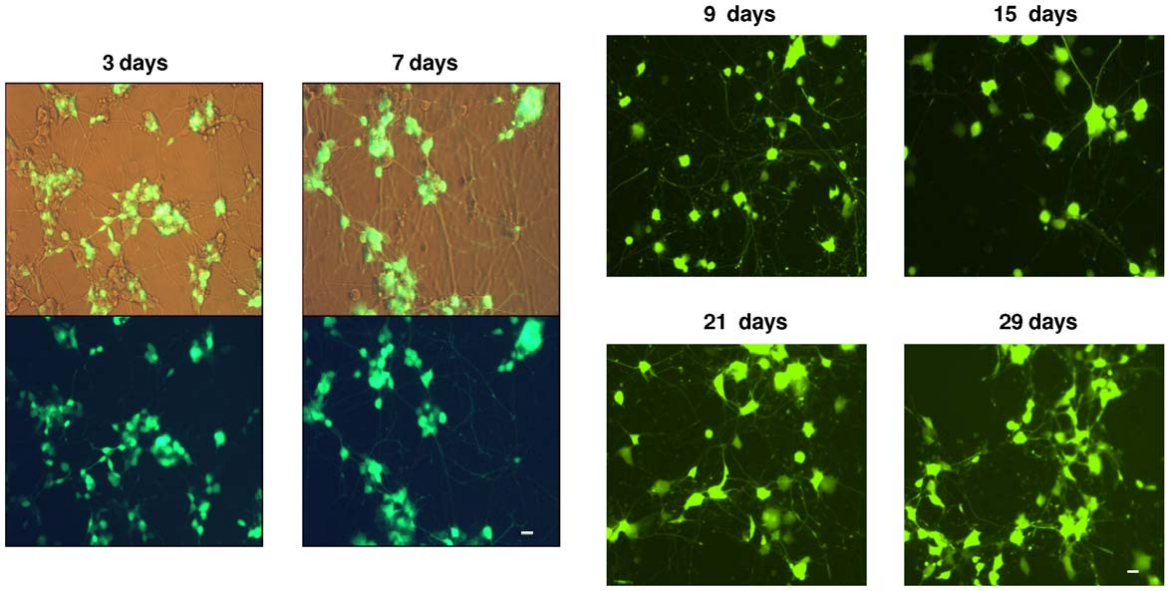
RT43.2GFP gammaretroviral vector transduction into differentiated PC12 cells: longevity of expression. PC12 cells were grown in 4-well Matrigel-coated chamber slides, treated with NGF for 5 days, and exposed to gammaretroviral vectors in the continuous presence of NGF. Representative examples of NGF-treated PC12 cells exposed to RT43.2GFP vectors were photographed with a 20× objective, 3 days and 7 days after exposure to viral vectors using phase contrast and a FITC filter. Representative fields of differentiated PC12 cells 9, 15, 21, and 29 days after exposure to RT43.2GFP retroviral vectors were photographed using a FITC filter. Scale bars = 20 µm.

Finally, we examined the persistence of GFP expression in transduced differentiated PC12 cells (Figure 5). GFP expression was maintained for up to one month in culture, consistent with stable expression from an integrated viral promoter, since the persistently strong GFP expression is seen well beyond both the known half-life of GFP itself [33] and the ability of unintegrated vector DNA to persist in transduced cells [34].

### Gammaretroviral mediated gene delivery of Bcl-xL rescues transduced PC12 cells from serum starvation- and/or NGF withdrawal-induced death

Having demonstrated efficient transduction of differentiated PC12 cells by MLV-based vectors bearing VSV envelope proteins we proceeded to assess whether MLV-based vectors could deliver and maintain expression of a transgene at levels sufficient to result in therapeutic benefit. It has been previously shown that cell death in PC12 cells can be induced by either serum or NGF withdrawal. Furthermore, cell death induced in differentiated PC12 cells by NGF withdrawal resembles that of NGF-deprived sympathetic neurons [35]. Blomer et al. [36] used lentiviral vectors encoding Bcl-xL, a protein that suppresses apoptotic cell death [37], [38], to assess whether death induced in differentiated PC12 cells by NGF withdrawal could be prevented by exposure of these cells to lentiviral vectors expressing Bcl-xL prior to NGF withdrawal. Lentiviral vector mediated expression of Bcl-xL in PC12 cells allowed cells to survive in the absence of serum or NGF [36]. We adopted this experimental paradigm to assess whether we could achieve similar results using gammaretroviral vector expressing Bcl-xL. The vector we employed encodes a fusion protein (GFPBcl-xL) previously shown to maintain wild type Bcl-xL biological function [38]. Infection of PC12 cells with RT43.2GFPBcL-xL vectors demonstrated the appropriate cytoplasmic redistribution of GFP expression to that associated with GFPBcl-xL protein distribution in transduced cells (Figure S2) and consistent with the previously reported distribution of Bcl-xL [38].

**Figure 6:**
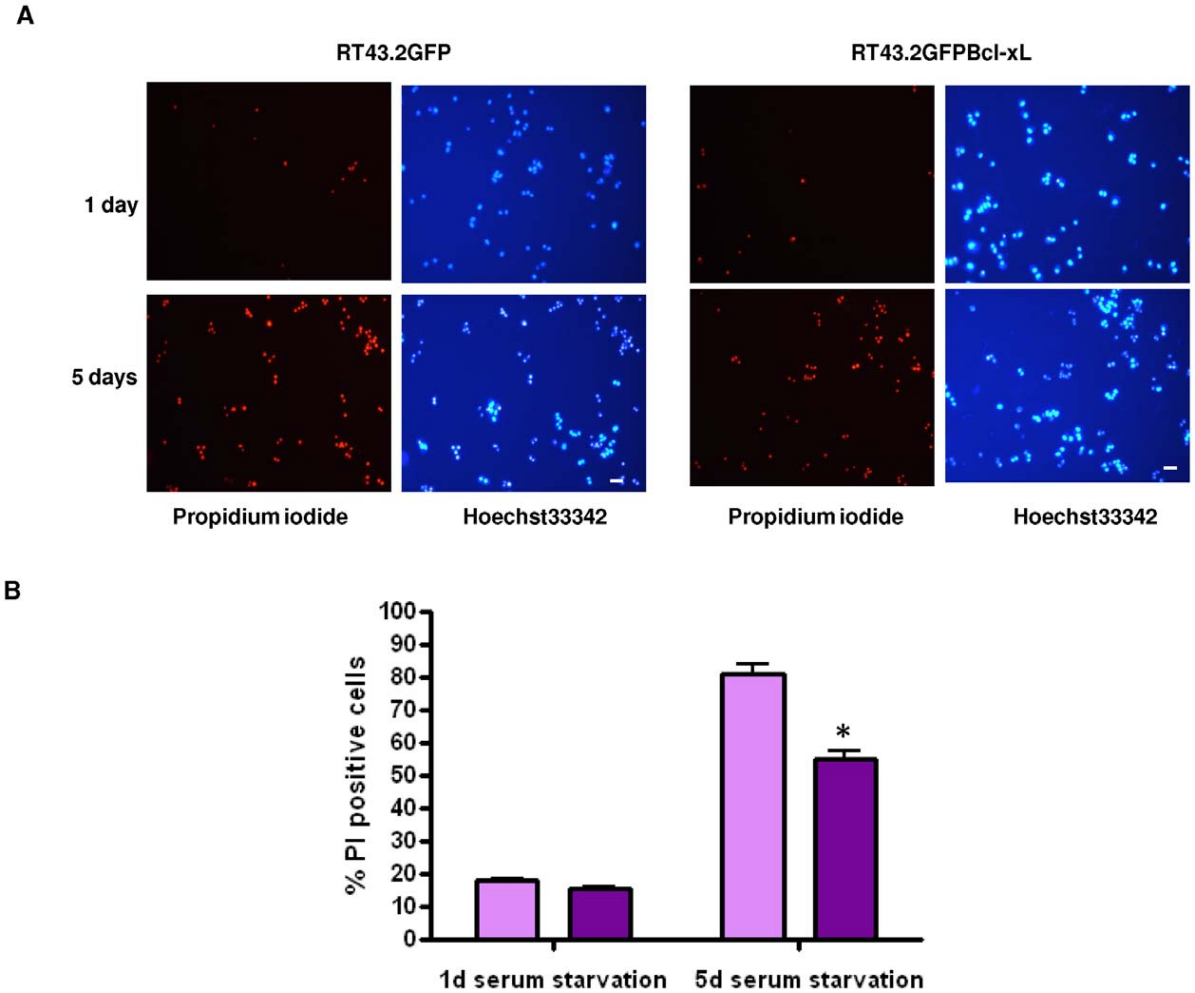
RT43.2GFPBcl-xL vector exposure decreased PC12 cell death induced by serum withdrawal. **A**. Undifferentiated PC12 cells on Matrigel-coated wells were transduced with control RT43.2GFP (left four panels) or RT43.2GFPBcl-xL vectors (right four panels) and then serum was withdrawn for either one day (upper panels) or 5 days (lower panels). After either one or five days propidium iodide was used to stain dead cells, and total cell count was obtained by Hoechst33342 staining. The cells were photographed with a 20× objective using either a rhodamine (PI) or DAPI filter. Scale bars = 20 µm. **B**. Propidium iodide (PI) stained dead cells and Hoechst33342 stained total cells were used to determine the effects of GFPBcl-xL expression (dark purple bar) compared to GFP expression alone (light purple bar) on cell viability. Percentage of PI positive cells versus Hoechst33342 stained total cells is represented by the mean value of several fields obtained from three experiments. *P<0.01.

Undifferentiated PC12 cells were exposed to control RT43.2GFP or RT43.2GFPBcl-xL vectors and then assessed for Bcl-xL-mediated survival effects after withdrawal of serum, in order to examine the anti-apoptotic properties of Bcl-xL delivered by gammaretroviral vectors. Seventy-two hours after PC12 cells were exposed to gammaretroviral vectors, serum was withdrawn, and cell death was assessed one and five days later. Expression of gammaretroviral vector-delivered Bcl-xL significantly decreased cell death induced by serum starvation after five days (Figure 6A, bottom panels). Quantification of propidium iodide (PI)-stained dead PC12 cells (Figure 6B) showed a statistically significant difference in the PC12 cells exposed to RT43.2GFPBcl-xL (55%) compared to those exposed to control vector (81%).

**Figure 7:**
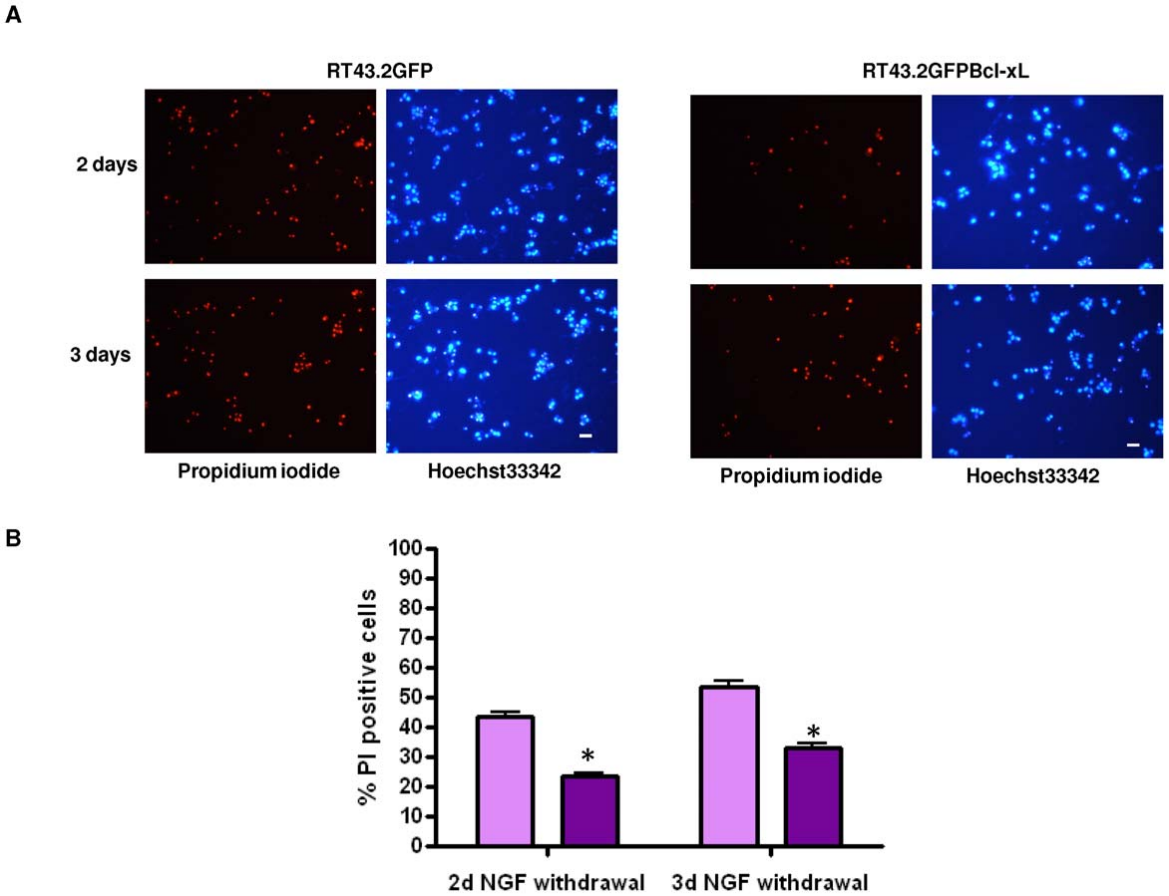
PC12 cells expressing Bcl-xL demonstrated reduced NGF-withdrawal induced death. **A**. Undifferentiated PC12 cells on Matrigel-coated wells were transduced with control RT43.2GFP (left four panels) or RT43.2GFPBcl-xL vectors (right four panels), differentiated with 100 ng/ml NGF for five days, and then NGF was withdrawn for either 2 (upper panels) or 3 days (lower panels) after which PI was used to stain dead cells and total cell count was obtained by Hoechst33342 staining. The cells were photographed with a 20× objective using either a rhodamine or DAPI filter. Scale bars = 20 µm. **B**. PI stained dead cells and Hoechst33342 stained total cells were used to determine the effects of GFPBcl-xL expression (dark purple) compared to GFP expression alone (light purple) on cell viability. Percentage of PI positive cells versus Hoechst 33342 stained total cells is represented by the mean value of several fields obtained from three experiments. *P<0.01.

In order to insure that retroviral vector mediated Bcl-xL expression was sufficient to rescue differentiated cells from cell death, PC12 cells were exposed to either RT43.2GFP or RT43.2GFPBclxL vectors, and treated with NGF for five days after which NGF was withdrawn. Substantial rescue from cell death after NGF withdrawal/serum starvation was observed at day two in PC12 cells exposed to vectors expressing Bcl-xL, compared to those exposed to control vector (Figure 7A). Two days after NGF withdrawal 23% of the cells exposed to RT43.2GFPBcl-xL were dead (PI stained) whereas 44% of cells exposed to control RT43.2GFP vectors stained with PI (Figure 7B). Three days after withdrawal, the effect was even more pronounced, with 33% of the cells dead following exposure to RT43.2GFPBcl-xL compared to 53% cell death for control cells (Figure 7B).

### Gammaretroviral mediated gene delivery of Bcl-xL to differentiated PC12 cells provided rescue from NGF-induced cell death

**Figure 8:**
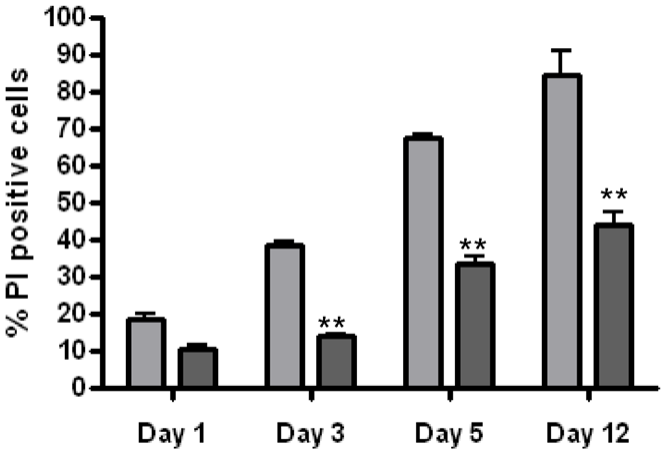
Differentiated PC12 cells exposed to RT43.2GFPBcl-xL vectors are rendered less susceptible to death attributable to NGF withdrawal. PC12 cells were plated on Matrigel-coated wells, and treated with 100 ng/ml NGF for five days. Differentiated cells were transduced with RT43.2GFP (grey) or RT43.2GFPBcl-xL (black) vectors during continued treatment with NGF. Three days after infection, the cells were washed with serum-free and NGF-free medium once and fed with serum-free and NGF-free medium for different intervals. PI and Hoechst33342 were added and the cells photographed using a 20× objective using either a rhodamine or DAPI filter. Percentage of PI positive cells versus Hoechst33342 stained total cells is represented by the mean value of several fields obtained from three experiments. *P<0.001.

The experiments depicted in Figures 6 and 7 show that Bcl-xL expression from a gammaretroviral transcriptional unit can protect both proliferating and growth-arrested PC12 cells from cell death triggered by serum or trophin withdrawal, but do not show that the gammaretroviral transcriptional unit, when introduced into post-mitotic cells, is capable of both efficient transduction and functionally relevant expression. To demonstrate this, PC12 cells were treated with NGF for five days prior to exposure to RT43.2GFPBcl-xL or RT43.2GFP retroviral particles, and subsequently maintained in NGF for an additional three days prior to challenge with NGF withdrawal. PC12 cell complete medium was then replaced with NGF- and serum-free medium. Differentiated PC12 cells exposed to RT43.2GFPBcl-xL vectors showed a significantly greater ability to survive at different days after serum and NGF withdrawal (Figure 8). Thus, RT43.2GFPBcl-xL transduction caused a significant decrease in the percentage of dead cells resulting from serum starvation and NGF withdrawal compared to control cells.

### Gammaretroviruses and gammaretroviral vectors infect rat cortical neurons

**Figure 9:**
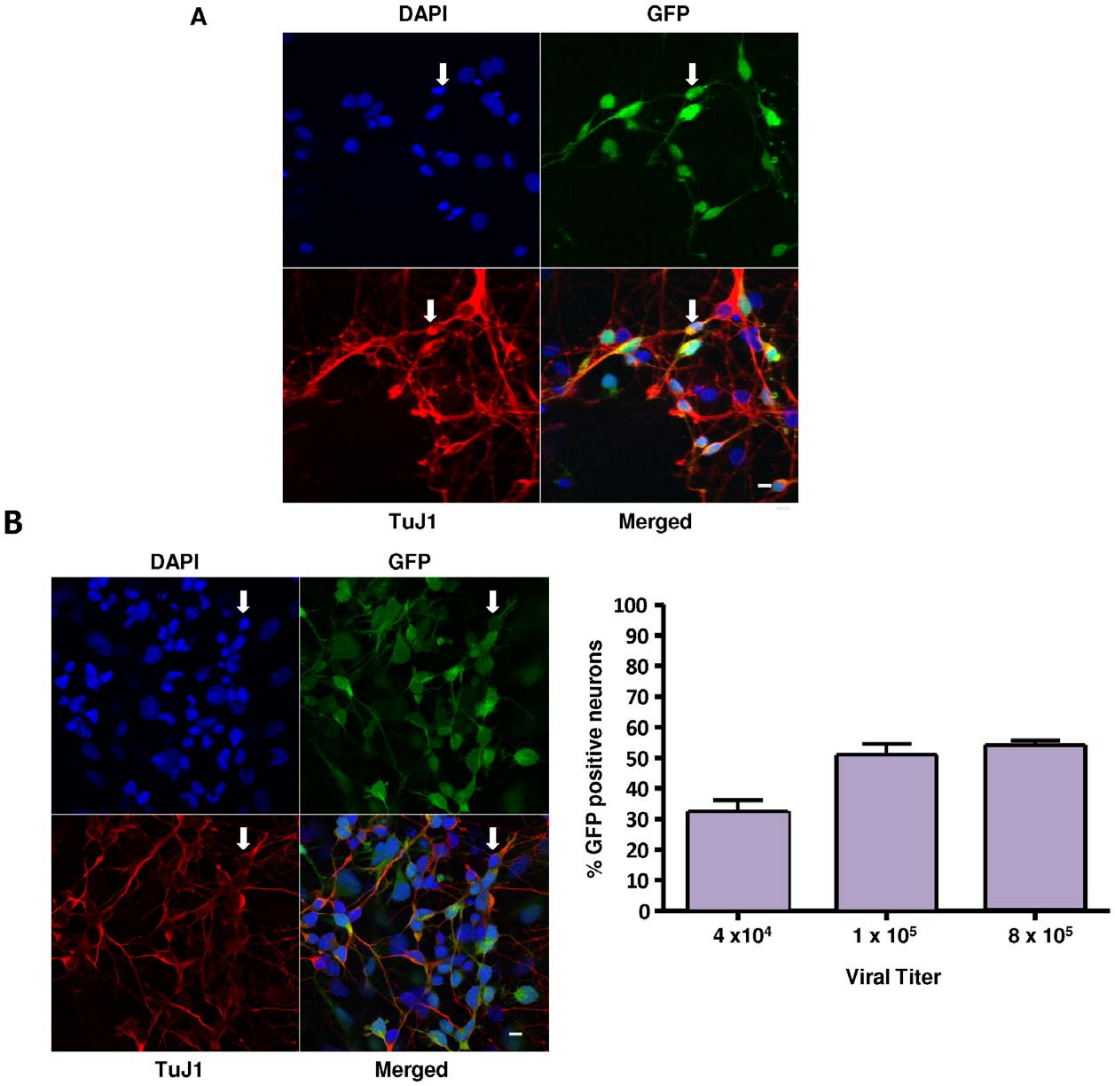
Rat cerebral cortical neurons are susceptible to infection by the gammaretrovirus, AZE-GFP, as well as RT43.2GFP gammaretroviral vectors. **A**. Cortical neurons were cultured from rat embryo brains at day 18 for seven days *in vitro* and then exposed to AZE-GFP in the presence of 1 µg/ml polybrene. Polybrene and virus were removed after 18 hours and cells immunostained with anti-GFP and TuJ1 antibodies, followed by DAPI staining, five days post-AZE-GFP exposure, and photographed at 63× with a laser scanning confocal microscope. Arrows indicate GFP-positive TuJ1 positive cells. Scale bar = 3 µm. **B**. Panel of representative confocal micrographs (left) of rat cerebral cortical neurons 5 days after exposure to RT43.2GFP vectors. Cells were immunostained with anti-GFP antibody and TuJ1 antibodies, followed by DAPI staining. The lower right hand quadrant of the panel shows the merged staining. Arrows indicate GFP positive, TuJ1 positive neurons. Scale bar = 3 µm. Graph of transduction efficiency (right) shows percentage of GFP-positive cells compared to total TuJ1 positive cells after exposure to 4×10^4^, 1×10^5^, and 8×10^5^ viral particles.

It was previously reported that cells must be mitotically active to enable infection by gammaretrovirues such as MLVs [4], [5]. These experiments were carried out using NIH3T3 cells that had been growth-arrested by serum withdrawal [4] or in rat fibroblast cell lines that had been mitotically arrested by withdrawing serum and adding aphidicolin [5]. Thus cortical neurons–post-mitotic neuronal cells that cannot divide in culture–would be expected to be resistant to infection by MLVs based on these previous findings. To assess whether this was the case rat cortical neurons were cultured in Neurobasal medium for seven days. Under these conditions, proliferating cells such as astrocytes, microglia, and the very small percentage of neuronal progenitor cells initially present in embryonic day 18 cortical tissue are gone, and post-mitotic, fully mature neuronal cells remain, with minimal (<1%) contamination by glial cells [39], [40]. Cultures were exposed to AZE-GFP and confocal microscopy was used to identify infected neurons. AZE-GFP (schematically depicted in Figure S1B) is an infectious, replication competent form of Moloney MLV containing an amphotropic MLV envelope, and an internal ribosomal entry site (IRES) adjacent to a GFP gene [41]. Five days after initial exposure to AZE-GFP particles, primary cultures of rat cortical neurons were stained with DAPI, a fluorescent stain that binds DNA to monitor the number of cell nuclei in a microscopic field, TuJ1 an antibody that detects neuron-specific class III β-tubulin, and a GFP antibody that stains cells expressing the GFP transgene. As shown in the merged image in Figure 9A, a substantial number of TuJ1 positive cells in rat cortical neuronal cultures were co-stained with GFP antibody (as shown by arrows). Thus, replication-competent MLV can infect rat cortical neurons.

**Figure 10:**
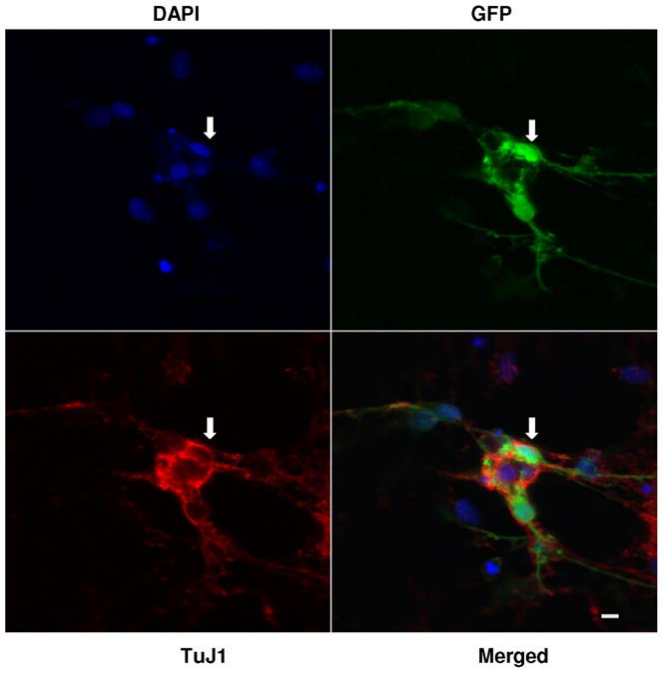
Transduction of lentiviral vectors into rat cerebral cortical neurons. Cortical neurons were cultured from rat embryonic brains at day 18 for seven days *in vitro* and then exposed to 9×10^5^ lentiviral vector particles, comparable to the highest MOI used for gammaretroviral transduction. Five days later, the cells were fixed and immunostained with anti-GFP and TuJ1 antibodies, followed by DAPI staining. The cells were photographed with a laser scanning confocal microscope at 63×. Arrows indicate GFP-positive, TuJ1 positive cells. Scale bars = 3 µm.

In addition to reports that growth-arrested cells were resistant to infection by replication competent MLVs it has also been previously shown that infectious but replication incompetent MLV-based retroviral vectors also fail to infect chemically growth-arrested cell lines [7], [42]. Rat cortical neurons were exposed to MLV RT43.2GFP vectors to obtain a quantitative assessment of transduction. RT43.2GFP vectors, in contrast to AZE-GFP virus, are not replication competent, therefore all GFP positive cells represent “initial hit” target cells rather than cells infected as a consequence of virus spread. A representative field of rat cortical neurons five days after exposure to RT43.2GFP vectors is shown in Figure 9B, left. Arrows depict TuJ1 positive cells that are also GFP positive and therefore correspond to transduced neurons. More than 50% of cortical neurons exposed to 8×10^5^ infectious vector particles per well are transduced (Figure 9B, right). It is important to note that the MOI used in our studies with retroviral vectors was significantly lower (viral titer of 8×10^5^ shown in Figure 9B corresponds to an MOI of 8) than that previously used to show growth-arrested cell lines are resistant to MLV retroviral vectors [7], [42] and thus unlikely to represent pseudotransduction i.e., the passive transfer of GFP. In fact, this gammaretroviral MOI is lower than that used in typical lentiviral transductions (MOIs around 20) in cultured rat cortical neurons reported by others [40]. In addition, development of fluorescence in transduced neurons, as in differentiated and undifferentiated PC12 cells, was gradual over days, definitively ruling out the possibility of pseudotransduction. Finally, we directly compared gammaretroviral and lentiviral vector transductions in cortical neurons. At an MOI of 9, lentiviral transduction of cortical neurons was about 50% (Figure 10), similar to that seen for gammaretroviral transduction of these cells.

## Discussion

In this report, we demonstrate that gammaretroviruses, and vectors derived from them, efficiently and stably transduce post-mitotic neurons and neuroendocrine cells. Our results contrast with a general consensus that gammaretorviruses are blocked from stable integration into proliferation-arrested cells including post-mitotic neural cells that possess an intact nuclear envelope membrane. Stepwise optimization of virus and vector transduction conditions in non-differentiated PC12 cells, differentiated PC12 cells, and finally in neurons, suggest that with all other factors corrected for, differences in the structural features of gammaretroviruses compared to lentiviruses are not themselves determinative for efficiency of transduction of post-mitotic cells. Rather, either type of virus can provide the minimal components necessary for transduction of cells without disruption of nuclear envelope integrity, i.e. in post-mitotic and/or growth-arrested cells. Specifically, improvements in gammaretroviral vector development have taken place since those initially used to conclude that gene transfer by gammaretroviral vectors occurs only in cells that are actively replicating at the time of transduction. These can be summarized as follows. Employing quantifiable reporter genes, such as GFP present in the AZE-GFP virus and RT43.2GFP vector, replaces the requirement to detect viral protein expression in productively infected cells [4], [5] or the use of selectable markers such as the G418^r^ gene employed in the previously reported gammaretroviral vector studies [6], [7]. The use of a GFP marker precludes the need for G418 selection as well as providing a quick, practical and sensitive means of quantifying transduced cells, via direct observation. Similarly, infection of neurons by AZE-GFP can be discerned by co-expression of GFP in concert with appropriate phenotypic markers; thus, identification of infected neurons in a mixed population of mitotically active and growth-arrested cells is unfeasible using conventional PCR, measurement of reverse transcriptase activity, or other viral infection detection methods, but can be achieved using a GFP marker with reasonable accuracy. Finally, the notion that gammaretorviruses and vectors do not infect non-dividing cells originally grew from experiments that demonstrated the inability of gammaretroviral vectors to infect cell lines that were growth arrested by chemical means [4], [5], [6], [7]. PA317 MLV vectors used in these studies contain amphotropic MLV (A-MLV) envelopes, which use the phosphate transporter SLC20A2 as a receptor [43], [44], [45], and the previously utilized replication competent ecotropic MLVs (E-MLV) use the cationic amino acid transporter MCAT-1 as a receptor [46]. It is worth considering the possibility that chemically induced growth arrest may down-regulate SLC20A2 and MCAT-1 surface proteins, resulting in an early block to A-MLV gammaretroviral vector entry and E-MLV entry.

Thus, for a variety of historically valid, but since superseded, methodological reasons, an appropriately rigorous scrutiny of the ability of gammaretroviral vectors to transduce non-dividing cells had not been previously undertaken since the initial reports indicated lack of infectivity of non-dividing cells by gammaretroviruses [6], [7]. Similar considerations apply to the assessment of the ability of gammaretroviral vectors to infect growth-arrested or non-dividing neuron and neuroendocrine cells [36]. The ability of gammaretroviruses to infect non-dividing cells has important implications, as it suggests that the tropism of other gammaretroviruses could be expanded to include cells previously thought to be resistant to infection.

While vectors based on MLV currently offer no intrinsic advantage over lentivirus-based vectors in transduction of post-mitotic cells, our results clearly show that MLV-based vectors can be employed to infect target cells previously considered to be resistant to non-lentiviral retroviral vector mediated gene transfer. We provide here evidence that gammaretroviral vector mediated gene delivery to non-dividing cells occurs, and is efficient enough to allow functional rescue of fully differentiated cells from neurotrophin withdrawal-induced death by an anti-apoptotic protein expressed from a retroviral vector. This suggests that gammaretroviruses should be re-evaluated as functional gene delivery tools for non-dividing cells. Proposals that gammaretroviral vectors encoding suicide genes be employed to selectively kill dividing tumor cells in the nervous system may also require re-evaluation of potential bystander effects on non-dividing cells [47]. Similarly, it may be appropriate to re-examine the assumption that the quiescent status of hematopoietic stem cells is a major obstacle to successful gammaretroviral vector mediated gene delivery to these cells [48], [49]. Finally, a broader range of cell types should now be considered as potential targets for gammaretroviral infection in the context of viral pathogenesis in diseases associated with gammaretroviral infection, such as prostate cancer and chronic fatigue syndrome [1], [2].

## Materials and Methods

### Ethics statement

Experiments with cultured cortical neurons from rat brain (see below) were carried out using protocols LCMR-14 and LCMR-15 for obtention of rodent tissue, which are current and fully approved by the Animal Care and Use Committee of the National Institute of Mental Health Intramural Research Program.

### Plasmid construction

The RT43.2GFP or RT43.2GFPBcl-xL vector genome contains two long terminal repeats (LTR), a CMV IE enhancer within the U3 region of the LTR, MLV splice acceptor (SA) and donor (SD) sequences, a packaging site and a GFP- or GFPBcl-xL- encoding gene, schematically depicted in Figure S1A. The pAZE-GFP plasmid is a biologically active clone obtained from the laboratory of Christopher Logg [50]. It contains LTRs and *gagpol* derived from Moloney murine leukemia virus and an envelope gene derived from amphotropic MLV. Adjacent to the A-MLV envelope is an IRES that permits expression of GFP, schematically depicted in Figure S1B. The pRRL-SIN-18 plasmid was a generous gift of Tom Dull (Cell Genesys, Foster City, CA) [51].

The G1 biosensor plasmid pHDHBc-tdimer2 was provided by Dr. A.T. Hahn (Stanford University School of Medicine, Stanford, CA). To create the HDHBc-tdimer2 retroviral expression vector the open reading frame consisting of the carboxy terminus of human DNA helicase B fused to tdimer 2 [21] was isolated by digesting the biosensor plasmid with EcoRI, filling in the 5′ cleavage site overhang with Klenow enzyme, and then digesting with HindIII. This fragment was then ligated to the pLNCX retroviral plasmid (Clontech, San Francisco, CA) previously digested with HindIII-Hpa1. The resulting plasmid was sequence confirmed.

### Production of AZE-GFP virus and LNCX-HDHBc-tdimer2 viral vectors, and production and titration of gammaretroviral and lentiviral vectors

AZE-GFP virus and retroviral vector particles were produced by transient transfection of 293T cells using Profection calcium phosphate transfection kit (Promega, Madison, WI). For AZE-GFP virus, 20 µg pAZE-GFP plasmid was transfected into 293T cells and supernatants were pooled at 48 and 72 hours to produce AZE-GFP virus that was subsequently combined, pre-cleared through 0.45 µm syringe filters, and stored at 4°C. Particles were then purified by ultracentrifugation through a 25% sucrose cushion at 135,500× g for 3 hours. The pellet containing retroviral particles was resuspended in phosphate buffered saline (PBS) to obtain a 500-fold concentrated retroviral stock, and aliquots were stored at −80°C. For LNCX-HDHBc-tdimer2 vectors, 293T cells were transfected with 10 µg pLNCX-HDHBc-tdimer2, together with 5 µg pVSV-G and 2.5 µg pMuLVgagpol. 48 hours after transfection, the supernatant was concentrated 16-fold over Amicon 100,000 MW cut-off filters (Millipore, Billerica, MA). Gammaretroviral vectors were produced by transient transfection of 293T cells using a three-plasmid system: 10 µg pRT43.2GFP or pRT43.2GFPBcl-xL plasmid, 2.5 µg MoMLV gagpol plasmid, and 5 µg VSV-G envelope plasmid. Lentiviral vectors were produced by transiently transfecting 293T cells with three plasmids: 10 µg pRRL-SIN-18, 6.5 µg pMoMLVgagpol and 3.5 µg pVSV-G. After collecting viral supernatants, the vectors were concentrated to a 500-fold stock as mentioned above. The titer of concentrated vector particles was determined by transducing MDTF cells with serial dilutions of retroviral stock as follows: MDTF cells were seeded at a density of 2.0×10^4^ cells per well in a 24-well plate and transduced 6 hours later. Sixteen hours after transduction, medium was changed. GFP-positive cells were counted 48 hours after transduction, and retroviral titer was obtained by dividing the number of GFP-positive cells by the volume of vector particles.

### Cell culture

Rat pheochromocytoma cells (the PC12 cell clone-derived line PC12-G was used in these studies) [52] were cultured at 37°C with 10% CO_2_ and grown in DMEM supplemented with 7% fetal cow serum, 7% horse serum, 20 mM HEPES, and 1% glutamate on Matrigel (BD Biosciences, San Jose, CA) coated 24-well plates, or 4-well Permanox® plastic chamber slides (Nunc, Naperville, IL). Differentiated PC12 cells were obtained by treating PC12 cells with 100 ng/ml 2.5S NGF (BD Biosciences) after PC12 cells were plated, and fresh NGF was added every two days.

Primary cultures of rat cortical neurons were prepared and cultured as previously described [53]. Briefly, the cortical neurons were obtained from dissociation of cerebral cortices from rat embryos at day 18 by trypsinization and trituration, followed by DNase treatment. The cortical neurons were then cultured on 8-well Lab-Tek borosilicate coverglass chamber slides (Nunc) coated with poly-D-lysine (Sigma-Aldrich, St Louis, MO), in 10% FBS for one day followed by replacing medium with B27/Neurobasal serum-free medium supplemented with 25 mM glutamate (Invitrogen, Carlsbad, CA).

### Production of LNCX-HDHBc-tdimer2-transduced PC12 cells and quantification of cell cycle phase

PC12 cells were transduced with LNCX-HDHBc-tdimer2 retroviral vector, and selected with 800 µg/ml G418 for two weeks. These cells were plated onto 4-well Permanox plastic chamber slides coated with Matrigel, treated with 100 ng/ml NGF for five days, fixed with 4% paraformaldehyde for 15 minutes, stained with 20 µg/ml DAPI, and photographed with propidium iodide and DAPI filters at 20× magnification. The untreated cells were also photographed in the same way. The cells with red fluorescent nuclear localization (G1 cells) or exclusion (S/G2 cells) were counted. The experiment was repeated twice, and cells in 10–20 fields in each experiment were counted.

### Genomic DNA isolation and PCR analysis

PC12 cells were treated with NGF for 5 days to allow complete differentiation, and then exposed to VSV-G-enveloped RT43.2GFP vectors for 72 hours before being collected for genomic DNA analysis. Genomic DNA was purified with the Promega Wizard® genomic DNA purification kit in accordance with the manufacturer's protocol. To eliminate any low molecular weight DNA contamination, genomic DNA was run on 0.8% agarose gel for 40 min, and DNA fragment with size >10 Kb was excised and purified with the QIAEX II Gel Extraction Kit (Qiagen, Valencia, CA). PCR analysis for the integrated GFP sequences was performed using FastStart Taq DNA Polymerase (Roche Applied Science, Indianapolis, IN). Approximately 75 ng of each genomic DNA sample was used as template for PCR. Primers for detection of the GFP band of 636 bp from provirus were: 5′-GGTGCCCATCCTGGTCGAGCTGGACG-3′ (sense) and 5′-CGAACTCCAGCAGGACCATGTGATCG-3′ (antisense). As a control for DNA integrity, a 1 kb band was generated with rat glyceraldehyde-3-phosphate dehydrogenase (GAPDH) primer pair: 5′-GGTGAAGGTCGGTGTGAACGG-3′ (sense) and 5′-CCAGGGTTTCTTACTCCTTGGAGGCC-3′ (antisense). The PCR program used to amplify genomic DNA started with an initial activation step of 95°C for 5 minutes, followed by 30 amplification cycles of denaturing at 94°C for 30 seconds, annealing at 65°C for 30 seconds, and extending at 72°C for 1 minute, and ended with a final extension at 72°C for 7 minutes. PCR products were visualized with ethidium bromide after electrophoresis in 0.8% agarose gel.

### Assessment of the neuroprotective effects of Bcl-xL delivered by gammaretroviral vectors in PC12 cells

PC12 cells were assessed for the ability of Bcl-xL to prevent cell death using three different assay systems. First, undifferentiated PC12 cells were exposed to retroviral particles and grown in PC12 complete medium for three days, washed twice with serum-free medium (DMEM medium, 20 mM HEPES and 1% glutamate) and then cultured in serum-free medium. At different intervals, the cells were stained with 1 µg/ml propidium iodide (PI) (Calbiochem, San Diego, CA) to detect dead cells, and 5 µg/ml Hoechst33342 (Invitrogen) to detect total cells. The randomly chosen fields were photographed at 20× using a Nikon Eclipse Ti microscope equipped with black-and-white CD camera, and the total and dead cells counted. Second, undifferentiated PC12 cells were exposed to vectors and treated with 100 ng/ml NGF for five days, and then underwent simultaneous withdrawal of serum and NGF for different periods of time, stained with PI and Hoechst33342, and photographed at 20×, after which the cells were counted. The third method of assessment was performed with PC12 cells being differentiated for five days in the presence of NGF and then exposed to viral vectors for three days in the continuous presence of NGF, followed by withdrawal of NGF and serum. At different day intervals after the withdrawal, the cells were stained with Hoechst33342 and PI, and the numbers of total and dead cells were assessed.

### Qualification and quantification of GFP-positive and total cortical neurons after gammaretroviral infection

Five days after exposure to AZE-GFP virus in the presence of 1 µg/ml polybrene or to gammaretroviral vectors, cortical neurons were fixed with 4% paraformaldehyde for 20 min, and blocked with goat serum. The following primary antibodies were used: AlexaFluor488-labeled rabbit polyclonal anti-GFP antibody (Invitrogen) at 1∶300 for GFP and monoclonal antibody TuJ1 (Covance, Berkeley, CA) at 1∶300 for specific neuronal staining. AlexaFluor594-labeled goat anti-mouse antibody (Invitrogen) at 1∶200 was used as the secondary antibody. The nuclei of cells were stained with 20 µg/ml DAPI. Confocal microscopy was used to photograph the images of neurons in randomly chosen fields, and the immunoreactive and total cells were counted.

### Statistical analysis

Student's two-tailed t-test was used for all statistical analysis.

## Supporting Information

Figure S1 **Schematic depiction of the replication-competent GFP-expressing proviral genome and the packageable retroviral vectors employed in these studies.** **A**. RT43.2GFP or RT43.2GFPBcl-xL integrated genome containing a packaging (Y) site that extends into gag coding region but lacks a 5′ATG and with a TAG stop codon at the end of the optimized packaging site. Genomes also contain genes encoding enhanced GFP (RT43.2GFP) or GFP fused to Bcl-xL (RT43.2GFPBcl-xL). **B**. AZE-GFP [50] is a biologically active MLV proviral genome containing MLV LTRs and gagpol, and an amphotropic MLV envelope gene. A gene encoding enhanced GFP driven by an IRES is located downstream of the env gene. **C**. RRL-SIN-18, is a self-inactivating lentiviral vector lacking the promoter region of U3. Details of its construction are described in Dull et al., 1998 [51].
(TIF)

Figure S2 **Intracellular distribution of GFP and GFP-Bcl-xL proteins observed in PC12 cells transduced with gammaretroviral vectors.** PC12 cells transduced with concentrated viral vector particles containing either the RT43.2GFP genome (**A**), or the RT43.2GFPBcl-xL genome (**B**). GFP is distributed throughout the entire cell, whereas GFP fused to the Bcl-xL protein is redistributed to cytoplasmic region consistent with Bcl-xL protein distribution [38]. Fluorescent images were taken on a Leica inverted microscope using iVision software at 20× magnification. Calibration bar = 50 µm. Enlarged inset areas are outlined with red boxes.
(TIF)
