# Supplementary figures and images for: The Host Range of Gammaretroviruses and Gammaretroviral Vectors Includes Post-Mitotic Neural Cells
Source: PLoS One. 2011 Mar 28;6(3):e18072. doi: 10.1371/journal.pone.0018072 (PMC3065480; doi:10.1371/journal.pone.0018072)

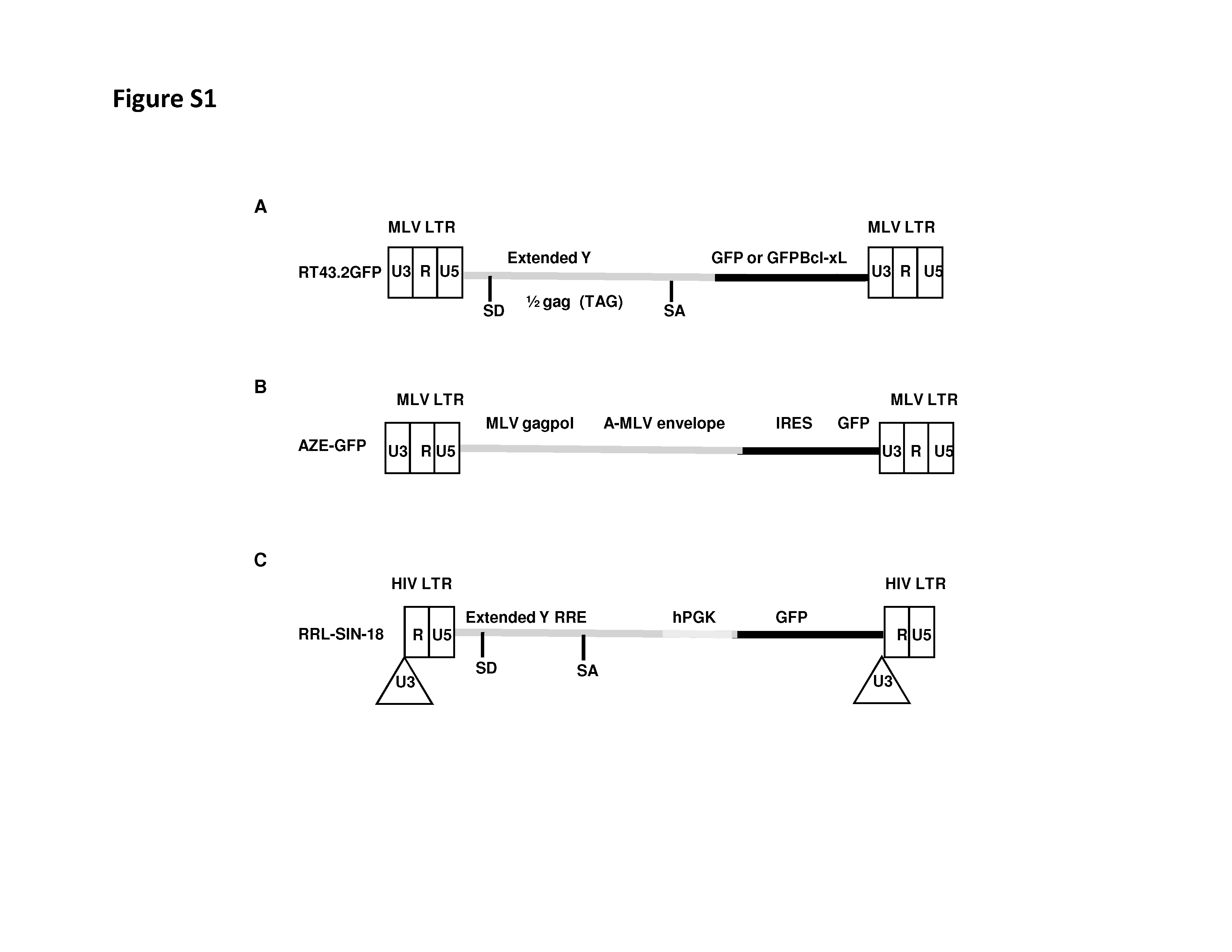

Supplement: Figure S1 — Schematic depiction of the replication-competent GFP-expressing proviral genome and the packageable retroviral vectors employed in these studies. A. RT43.2GFP or RT43.2GFPBcl-xL integrated genome containing a packaging (Y) site that extends into gag coding region but lacks a 5′ATG and with a TAG stop codon at the end of the optimized packaging site. Genomes also contain genes encoding enhanced GFP (RT43.2GFP) or GFP fused to Bcl-xL (RT43.2GFPBcl-xL). B. AZE-GFP [50] is a biologically active MLV proviral genome containing MLV LTRs and gagpol, and an amphotropic MLV envelope gene. A gene encoding enhanced GFP driven by an IRES is located downstream of the env gene. C. RRL-SIN-18, is a self-inactivating lentiviral vector lacking the promoter region of U3. Details of its construction are described in Dull et al., 1998 [51]. (TIF) [file pone.0018072.s001.tif]

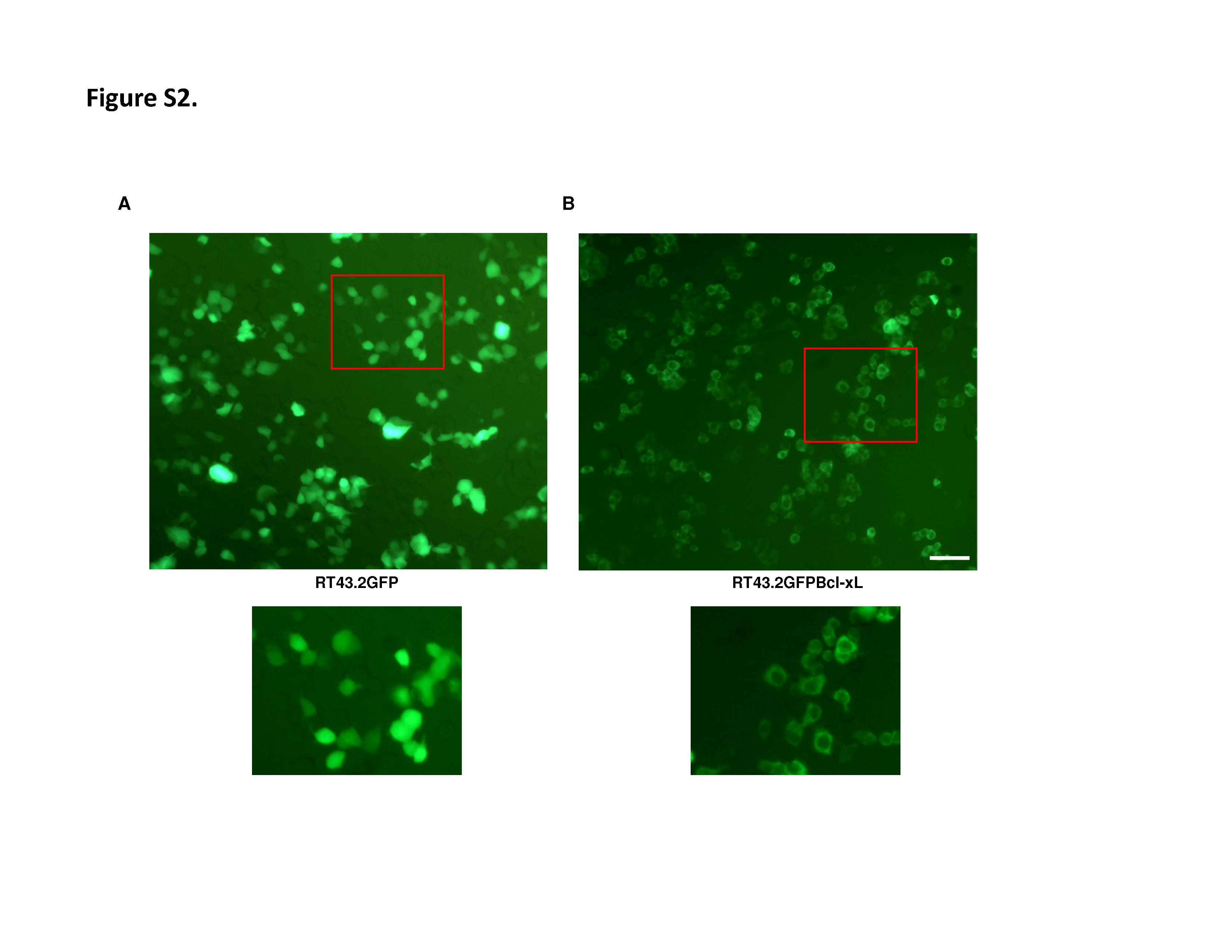

Supplement: Figure S2 — Intracellular distribution of GFP and GFP-Bcl-xL proteins observed in PC12 cells transduced with gammaretroviral vectors. PC12 cells transduced with concentrated viral vector particles containing either the RT43.2GFP genome (A), or the RT43.2GFPBcl-xL genome (B). GFP is distributed throughout the entire cell, whereas GFP fused to the Bcl-xL protein is redistributed to cytoplasmic region consistent with Bcl-xL protein distribution [38]. Fluorescent images were taken on a Leica inverted microscope using iVision software at 20× magnification. Calibration bar = 50 µm. Enlarged inset areas are outlined with red boxes. (TIF) [file pone.0018072.s002.tif]
